# Alkali-Activated Mineral Residues in Construction: Case Studies on Bauxite Residue and Steel Slag Pavement Tiles

**DOI:** 10.3390/ma18020257

**Published:** 2025-01-09

**Authors:** Lubica Kriskova, Vilma Ducman, Mojca Loncnar, Anže Tesovnik, Gorazd Žibret, Dimitra Skentzou, Christos Georgopoulos

**Affiliations:** 1 Department of Materials Engineering, KU Leuven, 3001 Heverlee, Belgium; 2 Slovenian National Building and Civil Engineering Institute, Dimičeva ulica 12, 1000 Ljubljana, Sloveniaanze.tesovnik@zag.si (A.T.); 3 SIJ Acroni d.o.o., Cesta Borisa Kidriča 44, 4270 Jesenice, Slovenia; 4 Geological Survey of Slovenia, Dimičeva ulica 14, 1000 Ljubljana, Slovenia; gorazd.zibret@geo-zs.si; 5 SE&C IKE, 11528 Athens, Greece; 6 Enalos Research and Development IKE, 15234 Athens, Greece

**Keywords:** alkali-activated materials, building materials, bauxite residue, steel slags

## Abstract

This research aimed to investigate the potential of using alkali activation technology to valorize steel slag and bauxite residue for the production of high-performance pavement blocks. By utilizing these industrial by-products, the study seeks to reduce their environmental impact and support the development of sustainable construction materials. Lab-scale testing showed that bauxite pavers showed a decrease in mechanical strength with increasing replacement of ordinary Portland cement. Partial replacement up to 20% still exceeded 30 MPa in compressive strength. Steel slag-based pavers achieved the 30 MPa threshold required for the application with selected mix designs. Pilot-scale production-optimized formulations and standards testing, including freeze–thaw resistance, confirmed the technical viability of these products. Life cycle analysis indicated a 25–27% reduction in CO_2_ emissions for slag-based tiles compared to traditional concrete tiles. Moreover, using industrial residue reduced mineral resource depletion. This study examined the properties of the resulting alkali-activated binders, their ecological benefits, and their performance compared to conventional materials. Through a comprehensive analysis of these applications, our research promotes the circular economy and the advancement of sustainable construction products.

## 1. Introduction

Growing industrialization and urbanization worldwide have led to a significant increase in the production of various industrial residues, posing both environmental and economic challenges. Efficient and sustainable methods for valorization are, therefore, essential in order to mitigate their environmental impact and harness their potential as valuable raw materials [1]. The residues addressed in this paper belong to the most prevalent and mass-produced industrial residues. Steel slag is a by-product of smelting (pyrometallurgical) ores or remelting steel scrap in an electric arc furnace. It is mainly a mixture of metal oxides and silicon dioxide and is the biggest by-product stream of the steel sector, accounting for 90% of the mass of by-product and waste generated. The amount of steel slag produced can vary depending on the type of steel being manufactured and the specific processes employed. Globally, steel slag production is estimated to range from 170 million to 250 million tons annually, as reported by Reddy et al. [2]. Although steel slag is highly recyclable, the European steel industry has focused its efforts on the improvement of by-product recovery and quality, resulting in being closer to its “zero-waste” goal [3]. Bauxite residue (BR) is an industrial waste generated during the processing of bauxite into alumina using the Bayer process. It is composed of a variety of oxide compounds, including iron oxides, which give it a red color. Over 95% of the alumina produced globally is through the Bayer process, where it is estimated that for every ton of alumina produced, approximately 1 to 1.5 tons of BR are also produced [4].

One promising approach for the valorization of steel slag and bauxite residue is through alkali activation. Inorganic polymers (geopolymers), being a subgroup of alkali-activated materials (AAM), are formed by the reaction of aluminosilicate materials with alkaline solutions, resulting in a three-dimensional network of Si-O-Al bonds [5]. This technology offers a sustainable alternative to traditional Portland cement by utilizing industrial by-products, reducing carbon emissions, and conserving natural resources [6,7,8].

Steel slag, with its high calcium content and suitable chemical composition, can be effectively utilized in the production of geopolymer pavement blocks, providing a sustainable solution for waste management while exhibiting superior mechanical properties and durability compared to conventional materials [9,10,11]. Research by Salman et al. [12,13] highlighted that alkali-activated binders based on steel slag can effectively replace Portland cement, providing similar or even superior mechanical properties. The activation process typically involves the addition of alkaline activators such as sodium hydroxide or sodium silicate, which initiate the polymerization of the slag components, forming a cohesive binding matrix. Wang’s research demonstrated that the incorporation of steel slag in alkali-activated systems significantly enhances compressive strength and durability compared to conventional cement [14]. Wu et al. investigated the hydration and microstructure of steel slag as a cementitious material, revealing that the heat release rate and total heat release energy could be indicative of the chemical reactivity of the slag [15].

The primary challenge in utilizing bauxite residue (BR) as the main raw material for AAM synthesis lies in its relatively low reactivity under alkali activation. This is despite its promising chemical composition, which includes aluminum (Al), silicon (Si), calcium (Ca), and iron (Fe), as well as its inherent alkalinity [16]. The crystalline nature of BR, as well as the complexity of the mineralogical composition, can limit its reactivity in AAM production, necessitating pre-treatment methods like mechanical milling to enhance its performance as a supplementary cementitious material [17,18]. The use of bauxite residue in the production of geopolymer concrete bricks has emerged as a promising avenue for recycling this material, contributing to sustainable construction practices [19]. Santi and ‘Ain [20] used BR as raw material for making concrete bricks without cement. Despite showing that BR has pozzolanic properties, they only achieved a compressive strength of 1.25 MPa. Nevertheless, bauxite residue can also be blended with other materials to enhance its properties. For example, the incorporation of fly ash with bauxite residue has been demonstrated to improve the mechanical performance and water resistance of the resulting composites [21]. This synergistic effect can lead to the development of new inorganic composite materials that are suitable for road construction and other applications. Research by Santi and ‘Ain demonstrated that bauxite residue can be effectively utilized in the manufacture of geopolymer concrete bricks, achieving compressive strengths greater than those produced with fly ash [21]. This indicates that bauxite residue not only serves as a viable alternative binder but also enhances the mechanical properties of the resulting concrete products.

The current paper goes beyond the existing literature by presenting two case studies that demonstrate the development of commercial-quality paving tiles using two distinct industrial residues with limited or no current valorization: bauxite residue and steel slag. Moreover, paving tiles were produced at a pilot scale and installed in public locations, serving both as demonstration units and real-life samples for continuous durability monitoring.

## 2. Materials and Methods

In this project, the bauxite residue (BR) used was provided by the Greek aluminum deposit area in Ag. Nikolaos Viotia, Greece. The as-received BR, d_90_ 45 μm, was deagglomerated in a planetary ball mill (HENAN 5.5 KW, 150 kg capacity, stainless steel balls 2–20 mm, Henan Lanphan Industry Co., Zhengzhou, China), and dried at 105 °C for 24 h. The mortars were prepared by blending the BR with Portland cement (CEM II/A-M (P-LL) 42.5 N, Lafarge, Heracles Group, Likovrisi, Greece) in various proportions (0–50%) and activated with 6 M NaOH solution (caustic soda flakes, 99% purity, S.C. Chimcomplex SA, Onești, Romania), maintaining a solution-to-powder mass ratio of 0.47. Standard limestone aggregates (sand 0–4 mm and coarse aggregates 4–16 mm) were used at an aggregate-to-powder mass ratio of 3.14. The mix design of lab-scale BR-based blocks, optimized to evaluate their performance, is outlined in Table 1. The mortars were cast in plastic (ertacetal) molds with high resistance to alkalis with dimensions (50 × 50 × 50) mm^3^. The entrapped air was removed with vibration for 2 min on an electrical vibration table. The specimens were demolded after 48 h and cured in a controlled environment (20 °C, 60% RH) until testing. After 28 days, the compressive and flexural strength of the cured specimens were measured according to EN 196-1 [22]. The leaching of heavy metals, especially chromium (Cr_tot_), was tested according to EN 12457-4:2002 [23]. Targeting the final application of the developed binder, the produced samples underwent the tests required by EN 1338:2003 [24] for concrete paving blocks, including the determination of total water absorption, the tensile splitting strength, the unpolished slip resistance value, and abrasion (according to the Böhme test) and freeze/thaw resistance with de-icing salt.

Based on the laboratory scale results, the mix design used for the production of tiles with dimensions (40 × 20 × 4) cm^3^ was adapted as follows. Portland cement was substituted by GGBFS (EcoCem, Dunkerque, France), and sodium silicate-based activation solution (8M NaOH and Na_2_SiO_3_ (molar ratio SiO_2_:Na_2_O < 3.4) at 50:50% *v*/*v* ratio) was used. The optimum solid-to-liquid mass ratio equaled 2.72 and the BR-to-GGBFS mass ratio was maintained at 1, whereas the aggregates-to-powder ratio equaled 0.82.

Two types of steel slag, i.e., EAF carbon slag (EAF C slag) and Ekominit S1 (a mineral product obtained by processing of a mixture of EAF stainless slag (EAF S slag) and ladle slag), were provided by SIJ Acroni, Slovenia. Within the optimization stage, the Ekominit S1 was blended with CEM I (52.5N), ground granulated blast furnace slag (GGBFS, EcoCem, France), and gypsum in various proportions and activated with sodium silicate (Betol 39T, Woellner, Ludwigshafen am Rhein, Germany). The molarity and concentration of the sodium silicate solution were adjusted by means of water and NaOH (97%, Sigma-Aldrich, St. Louis, MO, USA) addition. At a later stage, for the pilot production, the Ekominit S1 was blended with finely ground secondary copper slag (SCS) and GGBFS to serve as binder precursor. Meanwhile, the EAF C slag was used as an aggregate. To activate the mixture, a sodium silicate solution with a SiO_2_/Na_2_O molar ratio of 1.65 and 65 wt% water was used as the alkali activator. The mortars were mixed in a Hobart mixer according to the EN-196-1 [22] procedure and cast into (4 × 4 × 16) cm^3^ steel molds. The molds were covered with plastic foil to prevent moisture evaporation and placed in an oven at 60 °C. Mortar prisms were demolded after 24 h and further cured in a controlled environment (20 °C, 60% RH) until testing. Compressive and flexural strengths were determined using the 250 kN load cell and a crosshead speed of 2 mm/min (Instron 5985). Freeze–thaw resistance with and without the presence of de-icing salts was tested according to SIST 1026:2016 [25] and ASTM C666 [26], respectively, abrasion resistance according to EN 1338 [24], and leaching according to EN 12457-4:2002 [23].

## 3. Results

### 3.1. Chemical and Mineralogical Characteristics

The chemical composition of the residue used in this study is presented in Table 2. The BR used in the project was obtained from the deposit area with a moisture content of 8–12 wt%, density of 3.1 g/cm^3^, and d_25_, d_50_, and d_90_ of 1.1 μm, 2.3 μm and 49.8 μm, respectively. As for the steel slag (SS), the density equaled 3.3 g/cm^3^. In terms of particle size distribution, the SS exhibits the following parameters: d_25_, d_50_, and d_90_, which correspond to 47 μm, 234 μm, and 716 μm, respectively. The chemical composition of the materials, expressed in oxides, showed that SS and BR were primarily composed of CaO, SiO_2_, Fe_2_O_3_, and MgO, which collectively accounted for up to 84 wt%. CaO was the predominant oxide present in SS, unlike the BR, where Fe_2_O_3_ was the major oxide. The SCS was found to contain mainly Fe_2_O_3_, SiO_2_, and Al_2_O_3_, and the main oxides in GGBFS were CaO, SiO_2_, and Al_2_O_3_.

Both industrial residues were shown to be highly crystalline. BR was formed among others by hematite, diaspore, cancrinite, and kotoite. The SS consisted mainly of γ-C_2_S, β-C_2_S, merwinite, and periclase. The full list of mineral phases is provided in Table 3. It is noteworthy that both the GGBFS and SCS were more than 92% amorphous. The only mineral phase characterized in SCS was hercynite and quartz in GGBFS.

### 3.2. Lab-Scale Development and Performance Testing

**Figure 1:**
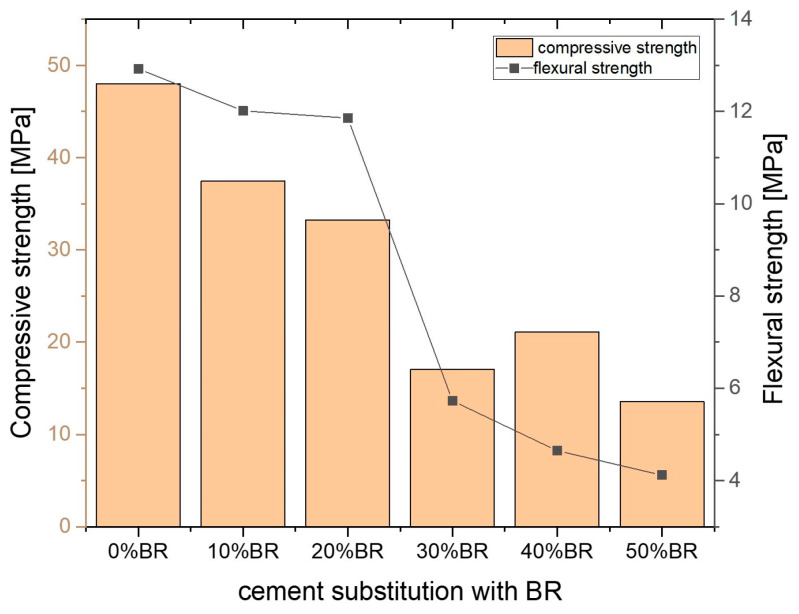
Compressive and flexural strengths of BR samples over 28 days.

The mix design for the BR-based alkali-activated tile was developed by progressively increasing the substitution level of OPC with BR, ranging from 0% to 50% by weight, and examining the impact of this substitution on compressive and flexural strength, as shown in Figure 1. Supplementary cementitious materials (SCMs) tend to have a slower reaction rate compared to OPC [27]; therefore, the strength levels were measured after 28 days only. A gradual decrease in compressive strength was observed with increasing OPC replacement. The strength dropped from 48 MPa in the pure OPC sample to 38 MPa and 32 MPa when 10% and 20% of OPC were replaced with BR, respectively, and continued to decrease to 12 MPa with 50% replacement. This indicates that the strength activity index (SAI) of the blended mortar was lower than that of pure OPC, indicating a negative impact of BR on strength development. A similar effect was observed with flexural strength, although the trend was less pronounced when 10% and 20% of OPC were replaced.

**Figure 2:**
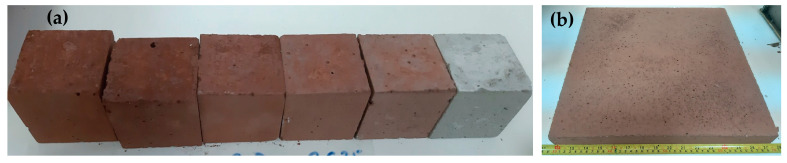
(**a**) Mortar cubes (50 × 50 × 50 mm^3^) with varying levels of cement substitution by BR for assessing the impact of this substitution; (**b**) BR-based alkali-activated paver (40 × 40 × 4 cm^3^).

Based on these results, it was decided to proceed with a 20% substitution of Portland cement for upscaling. Although the mechanical properties are slightly lower compared to the 10% substitution, this is offset by the higher amount of BR used in the formulation. Samples of dimensions 40 × 40 × 4 cm^3^ were prepared (Figure 2b) to evaluate their key characteristics, including water absorption, split resistance, and abrasion resistance, among others.

The initial phase of the SS-based mix design development and optimization process focused on parameters such as the use of OPC or GGBFS, the type of activating solution (silicate or hydroxide-based), and the liquid-to-solid ratio, among others. The parameters and their respective ranges are presented in Table 4 below.

**Figure 3:**
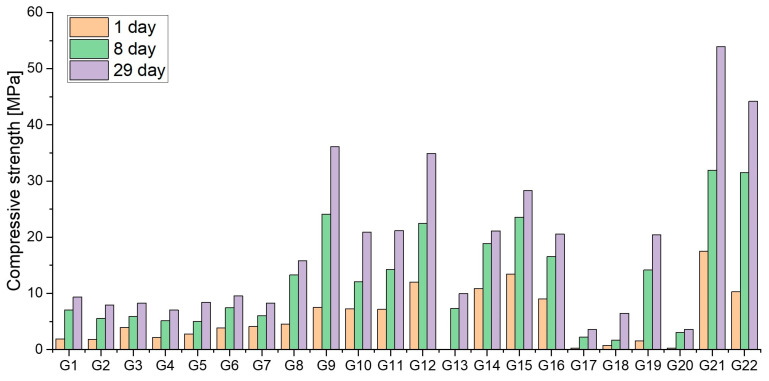
Compressive strengths of mortars with SS.

As in the case of the BR-based mix design, compressive and flexural strengths of SS-based samples were monitored to identify the most promising formulations (Figure 3). The optimal high compressive strength values were achieved with mix designs G9, G12, and G15. Both G9 and G12 exceeded the 30 MPa threshold required for the application. However, due to insufficient workability observed in the G12 sample, the G9 mix was selected for further evaluation of its properties, including porosity, freeze–thaw resistance, and more.

Additional tests were conducted on both the BR- and SS-based selected mix designs and BR-based pavers, with a 20% substitution of Portland cement and SS-based pavers for G9, respectively, and the results are listed in Table 5.

The testing of the BR-based mix design, following the EN 1338:2003 standard for concrete paving blocks, included evaluations of total water absorption, tensile splitting strength, unpolished slip resistance, abrasion resistance (Böhme test), and freeze–thaw resistance with de-icing salt. The results for the selected mix design fell within the acceptable range for commercially available precast concrete products.

The SS-based mix design performed well in terms of mechanical strength; it exhibited low freeze–thaw resistance and a notable reduction in flexural strength after freeze–thaw cycles. This issue was likely due to its high porosity of 21% [28]. Since the samples were not resistant to freezing in the presence of de-icing salt, they were unsuitable for use in environments exposed to salt, such as marine areas, roads, and sidewalks where de-icing salt is applied. Improvements were necessary for these conditions, along with further optimization to address leaching properties. The second round of optimization focused on samples G21 and G22. At this stage, the mix design shifted towards higher utilization of other byproducts, such as SCS, and involved the use of a stronger alkali solution, specifically a sodium- or potassium-based silicate solution with an SiO_2_/M_2_O ratio (M = Na/K) of 1.6–1.7 and a water content of 65–70 wt%. This optimization led to improved mechanical performance, achieving a compressive strength of 53 MPa and a flexural strength of 8 MPa after 28 days. These mix designs were used as starting points for large-scale tile production.

Another important parameter investigated was the leaching of heavy metals. This was for all heavy metals within permitted limits for the BR-based mix design, and the product can be classified, according to criteria in Council Directive 1999/31/EC on the landfill of waste [29], as “non-hazardous waste”. The leaching results for the SS-based mix design indicated slightly elevated levels of molybdenum (1.76 mg/kg) as well as the chromium (0.64 mg/kg) leached values, indicating that further optimizations were needed. The remaining tested parameters were found to be below the specified limit values as outlined in the directive.

### 3.3. Pilot-Scale Production and Performance Testing

To transition from lab-scale to pilot-scale production, several factors had to be considered, including the mix design scalability, pot life, and castability. Consequently, the mix designs developed at the laboratory scale were modified to meet these criteria. In the case of the BR-based tiles (dimensions: 1 × 0.5 × 0.02 m^3^), the cement was replaced with GGBFS, and a stronger activation solution was used, consisting of 8M NaOH and sodium silicate (SiO_2_/Na_2_O molar ratio < 3.4) in a 50:50% *v*/*v* ratio. The new optimal solid-to-liquid ratio was 2.72, with a BR-to-GGBFS ratio of 50:50% by weight. The 7-day compressive strength was measured at 58.8 MPa, with a flexural strength of 4.9 MPa and a density of 2.265 kg/m^3^.

For the SS-based tiles (dimensions: 40 × 40 × 4 cm^3^), the mix design was adapted by switching from a K-based activator to an Na-based one, adjusting the liquid-to-solid ratio, and adding a shrinkage-reducing agent to mitigate excessive drying shrinkage. The final mix designs used for pilot production are presented in Table 6.

The results of the leaching tests on BR tiles, presented in Table 7, indicate that no dangerous metals exceeded the regulatory limits. The produced BR-based tiles were tested according to EN 1339:2003/AC:2006 [30] (Table 8). The technical properties of the material produced from the BR were optimal and comparable with traditional products, such as pavers intended for walking surfaces. These pavers are particularly suitable for use on terraces, atriums, park areas, and other multi-purpose walking surfaces. For that purpose, high compressive strength, hydrophobic properties, and resistance to freezing and salt, as well as non-dangerous leaching and slip resistance, are some of the important factors. 

The produced SS-based tiles were tested based on the survey of standards for paving tiles. Results of the water absorption, freeze–thaw resistance, abrasion resistance, slip resistance, and leaching were also compared to selected commercially available concrete pavers tested in previous studies [31], as shown in Table 9.

The frost resistance of the tested alkali-activated pavers was evaluated through repetition of freeze–thaw cycles (30, 90, and 150), revealing no visible surface degradation or structural damage. This highlights the material’s durability to maintain its integrity in freezing environments, confirming its potential suitability for use in environments subjected to cyclic thermal stresses.

**Figure 4:**
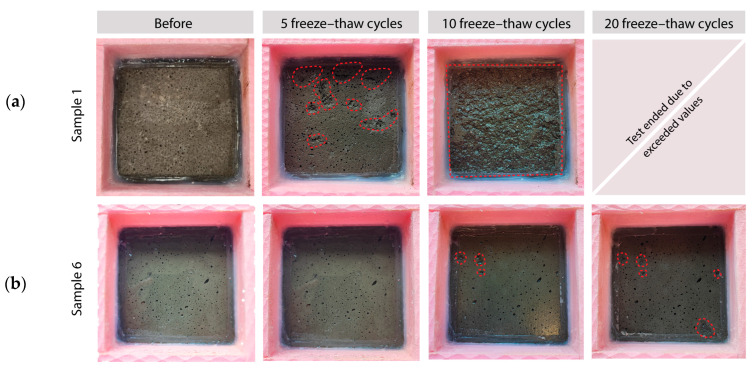
Freeze–thaw testing in the presence of de-icing salts for SS-based pavers: (**a**) pilot pavers before optimization showing surface scaling and (**b**) pilot pavers after optimization with significantly reduced scaling. Red dotted areas highlight spots of surface scaling.

The freeze–thaw resistance of the pavers in the presence of de-icing salts was initially unsatisfactory. The average cumulative mass loss per unit area after 10 was 3.2 mg/mm^2^, significantly exceeding the limits prescribed by SIST-TS CEN/TS 12390-9 [32] (Figure 4a). However, after further optimization of the material composition and production process, substantial improvement was achieved. The measured average cumulative mass loss per unit area was reduced to 0.01 mg/mm^2^ after 10 cycles and 0.03 mg/mm^2^ after 20 cycles (Figure 4b). Both values are well below the limit values, indicating a significant enhancement in the durability and resistance of the pavers to freeze–thaw conditions in the presence of de-icing salts. The improved resistance performance could be attributed to microstructural changes (porosity and an interfacial transition zone).

The skid resistance of the tested SS-based pilot pavers, measured under wet conditions, demonstrated an average Pendulum Test Value (PTV) of 64 across three directions, which is suitable for floor applications. The high skid resistance indicates the material’s excellent frictional properties, making it suitable in “end-use conditions”, such as wet public walkways. This value is well above the threshold typically required for safe floor applications, where the slip resistance value considered with a very low risk of slip injuries is more than 45 PTV in wet conditions, according to standard classification and technical building code recommendations [34,35]. The observed skid resistance can be attributed to the enhanced friction due to microstructural roughness and composition of the surface layer, affecting surface texture, aggregate exposure, and water drainage capacity [36,37].

While pavers are non-bearing construction elements, their durability under dynamic loads, such as pedestrian activity and environmental stressors (moisture and temperature fluctuations), is crucial for ensuring performance in various applications. Abrasion resistance, measured through groove length, provides an indicator of their suitability for demanding environments and prediction of long-term performance. The tested groove length of 15.6 mm after exposure to the wearing machine demonstrates improved abrasion resistance, suggesting that the material can withstand high-stress conditions. Besides the laboratory testing of pilot pavers, the long-term abrasion observation is held as demonstrative paved area (see Section 3.4 Demo installation).

While the integration of SS into pavers can enhance mechanical properties, durability, and industrial by-product valorization, it is necessary to examine leaching behavior. The leaching results for elements from the crushed SS-based tiles (according to the SIST EN 1744-3) were also improved compared to the original mix design, yet the leachate indicates slightly elevated molybdenum (0.9 mg/kg) leaching values (threshold limit 0.5 mg/kg), where the chromium (0.5 mg/kg) concentration reaches the threshold limit according to the Slovenian directive on the permissible content of pollutants in the product [38]. Further testing will involve the determination of the water permeability coefficient of SS-based tiles and performing leachability analysis of uncrushed tiles (according to the SIST EN 1744-3 [39] and additionally, SIST EN 16637-2 (tank leaching test) [40]). To address environmental safety, further strategies are considered to control leaching, like reducing porosity to minimize pathways for ion transport. This can be achieved through the addition of micro- or nano-additives, which can act as intact fillers or reactive secondary precursors. Nano- and micro-silica have been studied to enhance their microstructural properties, where mitigation of heavy metals leaching in AAMs was observed through densification of matrix, increased mechanical properties, and reduced porosity [41,42,43]. The addition of sorptive additives like zeolites or clay minerals might reduce the leachability of heavy metals. Svobodová et al. [44] demonstrated that alkali-activated zeolite foams possess a porous structure that facilitates the adsorption of heavy metals, significantly enhancing their immobilization. Similarly, protective coatings such as polymer sealants or hydrophobic treatments act as physical barriers, preventing water ingress and reducing the mobility of leachable ions. While effective, these solutions may involve additional material and energy costs, raising concerns about their economic and environmental sustainability. The process of AAM carbonation is a viable strategy that can address both concerns, by exposing the material to carbon dioxide (CO_2_) to form stable carbonate phases. As a result, densified matrix and reduced porosity can restrict the transport of leachable elements [45,46] or even physically encapsulate heavy metals, limiting their solubility and mobility [47,48].

### 3.4. Demo Installation

While laboratory scale testing can provide initial indicators of long-term durability (such as abrasion resistance), scaling up to small-scale production and conducting real-world observations of the materials outside the controlled laboratory environment is important.

**Figure 5:**
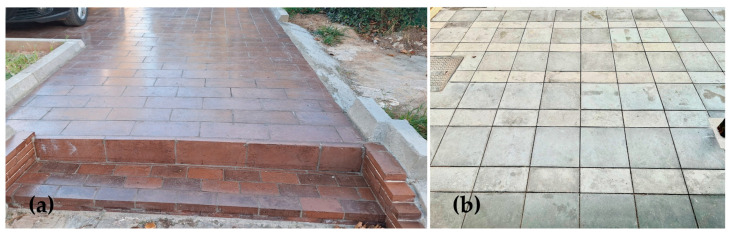
(**a**) The paved area of BR-based pavement blocks in Aspra Spitia, Greece; (**b**) the paved area of SS-based pavers at SIJ Acroni, Slovenia.

The BR pavement blocks were used for the paved part of a parking area in Aspra Spitia, Greece (Figure 5a), while 20 m^2^ SS-based pavers were laid down in April 2024 at the premises of SIJ Acroni, Slovenia (Figure 5b), where in addition to the demonstration, further long-term monitoring in real conditions will be performed. The laying of BR pavement blocks in the 250 m^2^ parking area was finished at the beginning of 2023 and since then, every six months, inspections on the performance are carried out. The demonstration site located in Aspra Spitia, Greece, is a coastal establishment that provides the opportunity to monitor the material’s behavior in a coastal environment. While no effects from sea mist have been observed thus far, ongoing monitoring will provide better long-term results on material behavior. Up to December 2024, no cracking or other deterioration on the installed blocks was observed, although the installation is used daily.

### 3.5. Life Cycle Impact Assessment of Developed Product

The LCA analysis, aligned with ISO standards [29,31,34], aims to evaluate a fire-resistant construction material (e.g., paving blocks, panels, tiles). The study considers two functional units: manufacturing a single 0.08 m^2^ SS-based tile (10.4 kg) and producing enough tiles to cover 1 m^2^ (130.4 kg).

**Figure 6:**
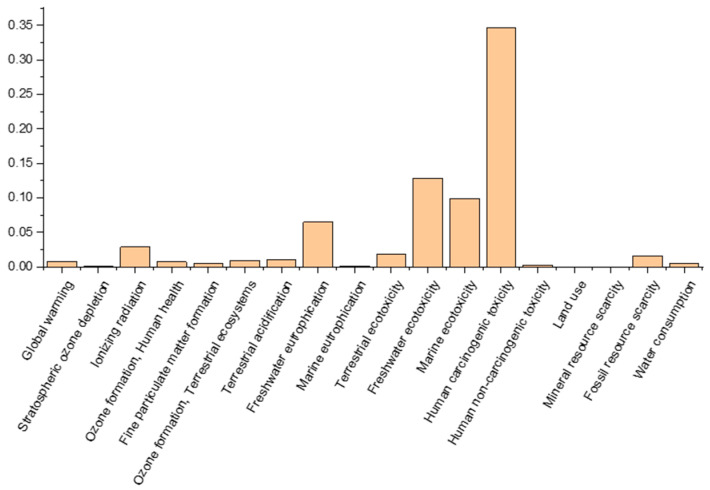
Environmental impact calculation around the production of one GEORIS tile (Method: ReCiPe 2016 Midpoint (H) V1.08/World (2010) H).

The cradle-to-gate scope includes raw material collection, manufacturing, and delivery but excludes usage and end-of-life stages. Background system data (e.g., grid energy and emissions) are separated from foreground data (e.g., data straight from the tile production). The final product was produced using the geopolymerization production process. Steps include slag collection, mixing, curing, and packaging. Figure 6 illustrates the impact that the production of one standard GEORIS tile has in the different environmental indexes.

All gathered data were imported into the software SimaPro version 9.5, which is a widely used commercial LCA software, alongside with the LCI database Ecoinvent12 version 3. The LCI results were evaluated through the ReCiPe (2016) method and the Recipe Hierarchist (H) perspective with the midpoint level method. The latter method was chosen to draw more conclusions and observe how the results are characterized by the different methods. The environmental performance of the SS-based tiles product was consistently described in a realistic way using the LCI data. As a result, energy, materials, and other resources needed and consumed during processing were used as inputs.

The LCA results for SS-based tile manufacturing are encouraging, particularly in terms of avoided environmental consequences. The manufacture of 7.76 kg, or 0.08 m^2^, of the SS-based tile demonstrates a significant amount of avoided impact, which may even cover for the negative consequences of the manufacturing process. The parameters were modified to suit the larger-scale industrial production process because the tile product was first developed at a laboratory scale before being upgraded in the KUL modular unit. Once the product is produced at full factory scale, it is anticipated that manufacturing times and total environmental impacts would decrease, pointing to a future production process that is more efficient.

**Figure 7:**
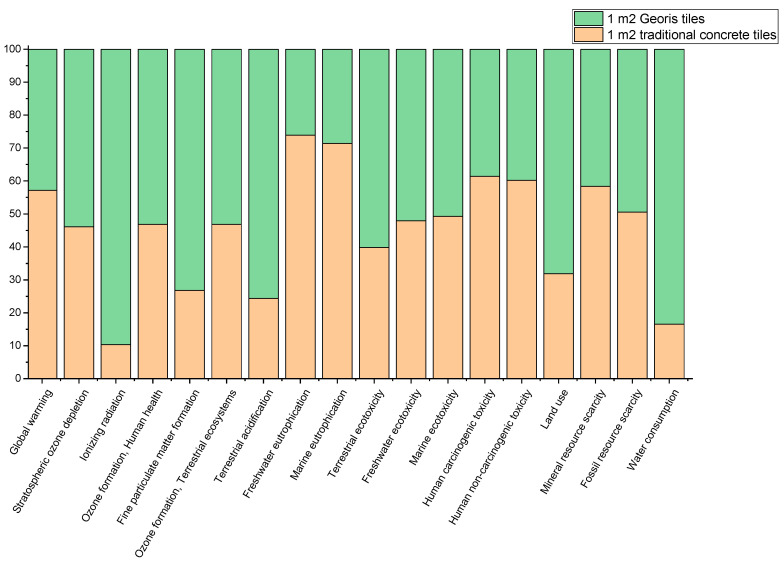
Comparison of impact factors of 1 m^2^ GEORIS paving block vs. 1 m^2^ traditional paving block in case scenario of the larger-scale industrial production process.

Comparing the two products and taking into consideration the worst scenario of landfilling of the industrial residues that were incorporated in GEORIS technology, Figure 7 shows that the concrete block has a higher human carcinogenic impact. This research highlights the potential benefits of applying GEORIS materials in construction, particularly in mitigating adverse health impacts. The SS-based tiles show a lower CO_2_ equivalent compared to traditional concrete tiles, indicating a reduction in carbon emissions (around 25–27%) for SS-based tiles per square meter. Additionally, GEORIS tiles have a positive effect on the eutrophication potential impact, with a 60% lower effect than concrete tiles, which is favorable in reducing water pollution and ecosystem degradation. Also, reasonably, geopolymer and alkali-activated tiles have a lower impact on mineral resource scarcity because those wastes are used from other industrial processes that, in the worst scenario, would be landfilled.

The hotspot analysis identified sodium hydroxide as the most contaminating input within the system boundaries examined. As part of the sensitivity analysis, an alternative scenario was considered where sodium hydroxide could be replaced by potassium hydroxide. The comparison between these two chemicals showed that sodium hydroxide has a higher negative environmental performance than potassium hydroxide. While substituting sodium hydroxide with potassium hydroxide could lower the overall environmental impact of GEORIS production, this change may come with added costs. Additionally, the GEORIS product would require further testing to ensure that performance standards are maintained with the use of potassium hydroxide.

### 3.6. Economic Analysis of Alkali-Activated Pavers

The economic assessment is based on the calculated costs of raw materials needed to produce 1 m^2^ of pavers. This price is then compared with the retail price of conventional concrete paving blocks.

In the case of SS-based tiles, two scenarios were considered (Table 10): the first one is a scenario without any optimization of mix design (“original mix design”), using raw materials with exact specifications as materials used to produce SS-based pavers as used for the SIJ Acroni demonstration field. The second scenario (“optimistic mix design”) shows the potential costs of locally supplied raw materials, which might have slightly different characteristics as those from the original mix design. In this case, prices for raw materials can be significantly lower, but the mix design might need to be slightly modified. The reason for the need of the “optimistic” scenario was that transportation costs across Europe for certain compounds in the original scenario make them unrealistically high, and local raw materials must be considered (optimization of recipe) in the case of potential future industrial production near the SIJ Acroni plant.

Compared with the retail price of classical concrete pavers, which is between 15–30 EUR/m^2^, it is evident that the cost of raw materials to produce alkali-activated pavers from SS, using the original components and recipe, is not economically viable, since the cost for raw material is around two times higher in comparison to the retail price of classical concrete pavers, without even accounting for the work, machinery, energy, and other indirect costs associated with such production. Finely ground quartz sand contributes around 50% of the total price of raw material for SS-based pavers in the original recipe, so the first potential way to improve economic performance is to replace this costly raw material with some less expensive one. In the terms of particle size distribution, the quartz sand M800 exhibits the following parameters: d_90_, d_50_, and d_10_, which correspond to 4.2 μm, 1.8 μm, and 0.4 μm, respectively. Since coarse aggregates can increase the fracture toughness of the matrix and impair tensile characteristics [49], the fine granulated quartz sand was used in the SS-based pavers. However, Jawaida et al. [50] used as a fine aggregate coarser sand, sand with a maximum particle size of less than 180 µm. In the optimistic scenario, it was assumed that granulated quartz sand of 0–180 µm from a local supplier could be used; however, recipe adjustments might be needed because these ingredients do not have the same characteristics as ingredients in the original recipe. Additionally, a local supplier of components needed for the activator was also selected in the case of the “optimistic” scenario. In the “optimistic scenario”, the cost for raw material is comparable to the retail price of classical concrete pavers, but without other costs like labor costs, energy, depreciation, etc.

Although Table 10 shows the price of raw materials only, other costs (i.e., energy, depreciation of machinery, labor costs, taxes, overhead, etc.) must be included in the final economic valorization. Initial estimations show promising results, especially if a local supplier is chosen and an optimization of the recipe using coarser and much cheaper quartz sand from the local supplier is foreseen. Economic performance can be improved if quartz sand can be replaced by a cheaper alternative, and even further improved in the case of higher CO_2_ taxes being imposed on cement producers in the future.

The analogous calculation of raw material prices for the alkali-activated pavers from the BR is shown in Table 11. It shows the retail prices of components needed to produce pavers for the test field. Calculations show that the raw materials price is between 25–30 EUR/m^2^. It must be noted that these prices in the case of any large-scale industrial production of pavers would be lower. Considering that costs of energy, depreciation, personnel, overhead, taxes, etc. are not considered and could add at least 20% to the final price, it can be concluded that with the current prices of concrete pavers, pavers from BR are not economically competitive. Data from Table 10 show that the costliest component for pavers from BR is activator, so any future attempts to improve their economic performance shall be focused on obtaining cheaper one. Nevertheless, it can be concluded that future economic optimization (i.e., lower costs of raw materials, optimized mix design, large-scale production, etc.) could make a low-CO_2_ BR paver a viable future alternative to the classic cement tiles.

## 4. Conclusions

Industrial by-products such as BR and steel SS demonstrate potential as valuable precursors for alkali-activated materials due to their chemical composition, which indicates sufficient levels of Si and Al necessary for the binding reaction. Despite the relatively high crystallinity of these industrial wastes, their amorphous phases actively participate in the alkali activation process, contributing to the formation of binding products. The crystalline phases, while largely inert in the reaction, act as functional fillers, reducing aggregate consumption and offering positive environmental benefits. Furthermore, the inclusion of these inert fillers can enhance material performance, improving abrasion resistance due to higher density and reducing shrinkage through the incorporation of sufficient aggregate content. These synergistic effects highlight the suitability of BR and SS for sustainable building material production.

By optimizing the mixtures and production parameters, it was possible to produce a product with comparable or even better mechanical and/or physical properties than commercially available products. The alkali-activated pavers from BR and SS exhibit parameters similar to commercial concrete pavers, including excellent freeze–thaw resistance, high skid and abrasion performance, and enhanced durability under challenging conditions. While the leaching of certain elements (Mo, Cr) requires further mitigation in SS-based pavers, future strategies such as carbonation not only address environmental safety but also align with economic and environmental policies targeting emissions reduction and net-zero industry goals. Additionally, calcium carbonate crystals that formed during carbonation can fill the pores and reduce the porosity, consequently enhancing resistance of the pavers to freeze–thaw conditions in the presence of de-icing salts.

LCA highlights the environmental advantages of SS-based tiles over conventional cement tiles. The comparison depicted significant reduction in CO_2_ emissions (25–27% per m^2^) and lower eutrophication potential impact (~ 60% lower), which contributes to reduced water pollution and ecosystem degradation. The input and usage of industrial waste as repurposed materials have value in the minimization of mineral resource scarcity, which otherwise would be landfilled. Therefore, a lower environmental footprint is achieved, and also new innovative and sustainable products are suggested. For further development of the GEORIS technology and products, continuous research is mandatory, from alternative inputs to process optimization, to enhance the environmental performance.

Economic estimates of small-scale laboratory production show that BR-based or SS-based tiles could compete with standard concrete products available on the market. However, careful cost optimization of pilot production and recipe adjustment, using locally supplied raw materials, is required to achieve economic competitiveness compared to concrete-based products. However, in the case of higher costs for CO_2_ emissions in the future, the production of alkali-activated products will even become more economical.

To address environmental safety and economic viability, further optimization of SS-based pavers will be performed before large-scale implementation. The strategy will involve carbonation of steel slag, which might reduce the leachability and could enhance resistance of the pavers to freeze–thaw conditions in the presence of de-icing salts. By using locally supplied raw materials, economic competitiveness compared to concrete-based products could be achieved. The substituting of sodium hydroxide with potassium hydroxide could lower the overall environmental impact of SS-based pavers; however, it might increase production costs. This will be applied only if it is economically viable.

Emphasizing the practical steps needed to translate lab-scale research into real-world applications by producing AAM pavers on a small scale aims to bridge the gap between lab-scale development and practical application by advancing the technology readiness levels (TRLs) to showcase future industrial feasibility and implementation. The demonstrational installation of pavers will provide further real-world observations of the materials under actual environmental conditions.

## Figures and Tables

**Table 1:** The BR-based mix design for lab scale, with provided mass in grams (g) for each component.

Bauxite Residue (45 μm)	Portland Cement (42.5 N)	Sand (0–4 mm)	Aggregate (4–16 mm)	6 M NaOH
**-**	280	330	550	132
28.0	252.0	330	550	132
56.0	224.0	330	550	132
84.0	196.0	330	550	132
112.0	168.0	330	550	132
140.0	140.0	330	550	132

**Table 2:** The chemical composition of residues presented in wt%.

Oxide	BR	SS	EAF-C	GGBFS	SCS
LOI (950 °C)		4	0.1		
SiO_2_	10	17	8	30	25
Al_2_O_3_	19	9	5	12	8
CaO	11	36	30	43	3
MgO	<1	13	10	7	1
K_2_O	<1	-	<1	1	-
Fe_2_O_3_	45	10	32	-	56
MnO	-	2	5	-	-
TiO_2_	6	<1	<1	-	-
Na_2_O	5	-	<1	-	-
SnO_3_	-	-	-	2	-
ZnO	-	<1	-	-	6
Cr_2_O_3_	-	3	2	-	-

**Table 3:** The mineral phases of the as-received residues, expressed in wt%.

Phase	Formula	BR	SS
Hematite/magnetite	Fe_2_O_3_/Fe_3_O_4_	33.8/-	-/2.6
Goethite	FeO (OH)	7.8	-
Diaspore/boehmite	α-AlOOH/γ-AlOOH	17.1/1.8	-
Bayerite/gibbsite	Al(OH)_3_	1.7/0.9	-
Nordstrandite	Al(OH)_3_		8.9
Cancrinite	Na_6_Ca_2_ (AlSiO_4_)_6_(CO_3_)_2_	12.6	-
Katoite	Ca_3_Al_2_(SiO_4_)_1.5_(OH)_6_	11.4	-
Calcite	CaCO_3_	7.9	-
Perovskite	CaTiO_3_	3.3	-
Quartz	SiO_2_	0.7	-
Anatase	TiO_2_	0.6	-
Brownmillerite	Ca_2_(Al,Fe)_2_O _5_	-	2.7
Periclase	MgO		13.2
β -C_2_S/γ-C_2_S	Ca_2_SiO_4_	-	12.6/21.6
Merwinite	Ca_3_Mg(SiO_4_)_2_	-	20.9
Bredigite	Ca_7_Mg(SiO_4_)	-	8.5
Portlandite	Ca(OH)_2_	-	2
Mayenite	12CaO·7Al_2_O_3_	-	7.1

**Table 4:** Optimization parameters for SS-based binder. Optimization range and selected mix designs (G9, G12, and G15) included due to their optimal compressive strengths.

	Optimization Range	G9	G12	G15
Ekominit S1	11–20 wt%	11.1 wt%	11.8 wt%	12.3 wt%
Cement	0–7 wt%	-	-	-
GGBFS	0–11 wt%	11.1 wt%	11.8 wt%	9.9 wt%
Gypsum	0–1 wt%	-	-	-
Molarity of NaOH solution	0–6	3 M/8.9 wt%	3 M/3.1 wt%+ 6 M/5.8 wt%	3 M/8.9 wt%
Na-silicate in the solution	0 and 11 wt%	2.2 wt%	2.2 wt%	2.2 wt%
Standard sand	65–67 wt% g	67 wt%	67 wt%	67 wt%
Powder-to-solution mass ratio	0.47–0.5	0.5	0.47	0.5

**Table 5:** The test results, in accordance with the EN 1338:2003 [24] standard for concrete paving blocks, are presented for two paving compositions: bauxite hybrid inorganic polymer paving blocks (left) and SS-based alkali-activated blocks (right).

BR-Based Mix Design		SS-Based Mix Design	
Total water absorption	5.8% (<6%)	Porosity	21%
Tensile splitting strength	4 N/mm^2^	Flexural strength—after 300 cycles	2.4 MPa
Unpolished slip resistance	USRV_wet_ 62.4 SRV_dry_ 64.5	Freeze–thaw resistance	No visual damage after 300 cycles
		Flexural strength	3.7 MPa
Abrasion	6553.2 mm^3^	Abrasion	19.3 mm
Freeze/thaw resistance with de-icing salt	0.4 kg/m^2^	Freeze/thaw resistance with de-icing salt	3.9 mg/mm^2^

**Table 6:** Mix designs of the large format tiles, in wt%.

BR-Based Mix Design		SS-Based Mix Design	
GGBFS	23.0	Ekominit S1	11.5
BR	23.0	SCS	11.5
8M NaOH	8.0	GGBFS	4.1
Sodium silicate (SiO_2_/Na_2_O MR~3.4)	8.0	M800-fine quartz sand	8.0
Limestone sand (0–2 mm)	16.0	EAF slag	51.6
Limestone gravel (2–6 mm)	16.0	1.65 NS 65	13.0
		Shrinkage-reducing agent	0.4
Solid/liquid	2.72	Binder/liquid	2.08

**Table 7:** Leaching test results on alkali-activated BR tiles.

Parameter	LoQ (mg/kg)	BR-Based Tile (mg/kg)
Ba	0.01	<LoQ
AS	0.01	<LoQ
Cd	0.005	<LoQ
Cr^tot^	0.05	<LoQ
Cr^+6^	0.05	<LoQ
Cu	0.1	<LoQ
Hg	0.0002	<LoQ
Mo	0.01	<LoQ
Ni	0.01	<LoQ
Pb	0.02	<LoQ
Sb	0.01	<LoQ
Se	0.001	<LoQ
Zn	0.5	<LoQ
Cl^−^	50	390
F^−^	1	5.5
SO_4_^−2^	100	660
Ba	0.01	<LoQ

**Table 8:** Large format BR tiles tested according to EN 1339:2003/AC:2006 Concrete paving slabs—requirements and test methods [30].

Tested Property	Obtained Value
Density	2.6 kg/m^3^
7-day compressive strength—EN 196-1 [22]	58 N/mm^2^
Total water absorption	12.4
Tensile splitting strength	4.9 N/mm^2^
Unpolished slip resistance value	USRV_wet_ 37 SRV_dry_ 81
Abrasion	2052 mm^3^
Freeze/thaw resistance with de-icing salt	1.1 kg/m^2^

**Table 9:** Properties of SS-based tiles from pilot production compared to the reference commercial products.

Testing Parameter	Standard	Result	Reference Concrete Product [31]
Freeze–thaw resistance	ASTM 666 [26]	No visual change after 150 cycles	Non-resistant
Freeze–thaw resistance in the presence of de-icing salts	SIST-TS CEN/TS 12390:9 [32]	0.01–0.03 mg/mm^2^	Non-resistant
Skid resistance	SIST-TS CEN/TS 16165:2016, Annex C [33]	64 PTV	69 PTV
Abrasion resistance	EN 1338 [24]	15.5 mm	22.1 mm

**Table 10:** Costs of raw materials for the production of 1 m^2^ of SS-based tiles using two scenarios.

		Original Recipe			Optimistic Scenario		
Material	Required Quantities of Raw Materials (kg/m^2^)	Cost of Raw Materials (EUR/t)	Potential Supplier	Cost of Raw Materials (EUR/m^2^)	Cost of Raw Materials (%)	Potential Supplier	Cost of Raw Materials (EUR/m^2^)
SS	13.6	0	Acroni	0	0	Acroni	0
SCS	13.6	77	Aurubis	1	77	Aurubis	1
GGBFS	4.8	236	ECOCEM	1.1	236	ECOCEM	1.1
Sand (M800)	9.5	1850	Agitrade	17.5	500	Termit	4.7
EAF C slag	60.9	0	Acroni	0	0	Acroni	0
Activator	15.4	1376	Kefo, ECP	21.1	486	Silkem, ECP	7.5
2 methyl	0.7	3520	Chamatek	2.5	3520	Chamatek	2.5
TOTAL PRICE of raw materials (EUR/m^2^)				43.3			16.9

**Table 11:** Costs of raw materials for the small-scale production of 1 m^2^ of pavers from BR.

	Required Quantities for 1 Panel (50 cm × 50 cm × 4 cm) (kg)	Required Quantities for 1 m^2^ (kg)	Component Price (EUR/kg)	Component Price (EUR/m^2^)
Bauxite residues	2.44	9.76	0.05	0.49
GGBFS	1.83	7.32	0.08	0.59
Metakaolin	0.31	1.24	0.16	0.2
River sand	3.05	12.2	0.01	0.06
Fibers	0.1	0.4	4.2	1.68
Activator	3.67	14.68	1.5	22.02
Surfactant	0.04	0.16	9	1.44
TOTAL:				26.47

## Data Availability

The original contributions presented in this study are included in the article. Further inquiries can be directed to the corresponding author.
